# Use of Virtual Reality Working Memory Task and Functional Near-Infrared Spectroscopy to Assess Brain Hemodynamic Responses to Methylphenidate in ADHD Children

**DOI:** 10.3389/fpsyt.2020.564618

**Published:** 2021-01-21

**Authors:** Sooah Jang, JongKwan Choi, Jooyoung Oh, Jungyeon Yeom, Narae Hong, Narae Lee, Joon Hee Kwon, Jieun Hong, Jae-jin Kim, Eunjoo Kim

**Affiliations:** ^1^Institute of Behavioral Science in Medicine, Yonsei University College of Medicine, Seoul, South Korea; ^2^OBELAB Inc, Seoul, South Korea; ^3^Department of Psychiatry, Yonsei University Gangman Severance Hospital, Seoul, South Korea; ^4^Department of Neurology, Yonsei University College of Medicine, Seoul, South Korea; ^5^College of Medicine, Hallym University, Chuncheon, South Korea; ^6^Department of Psychiatry, National Health Insurance Service Ilsan Hospital, Goyang, South Korea

**Keywords:** virtual reality, fNIRS, ADHD, working memory, n-back, Methylphenidate, mPFC

## Abstract

Virtual reality (VR) neuropsychological tests have emerged as a method to explore drug effects in real-life contexts in attention deficit hyperactivity disorder (ADHD) children. Functional near-infrared spectroscopy (fNIRS) is a useful tool to measure brain activity during VR tasks in ADHD children with motor restlessness. The present study aimed to explore the acute effects of methylphenidate (MPH) on behavioral performance and brain activity during a VR-based working memory task simulating real-life classroom settings in ADHD children. In total, 23 children with ADHD performed a VR n-back task before and 2 h after MPH administration concurrent with measurements of oxygenated hemoglobin signal changes with fNIRS. Altogether, 12 healthy control (HC) subjects participated in the same task but did not receive MPH treatment. Reaction time (RT) was shortened after MPH treatment in the 1-back condition, but changes in brain activation were not observed. In the 2-back condition, activation of the left dorsolateral prefrontal cortex (DLPFC) and bilateral medial prefrontal cortex (mPFC) was decreased alongside behavioral changes such as shorter RT, lower RT variability, and higher accuracy after MPH administration. Bilateral mPFC activation in the 2-back condition inversely correlated with task accuracy in the pre-MPH condition; this inverse correlation was not observed after MPH administration. In ADHD children, deactivation of the default mode network mediated by mPFC reduced during high working memory load, which was restored through MPH treatment. Our results suggest that the combination of VR classroom tasks and fNIRS examination makes it easy to assess drug effects on brain activity in ADHD children in settings simulating real-life.

## Introduction

Attention-deficit/hyperactivity disorder (ADHD) is one of the most common neurodevelopmental disorders in children. ADHD is defined by age-inappropriate symptoms of inattention, impulsivity, and hyperactivity. Patients with ADHD exhibit complex multisystem impairments in fronto-cingulo-striato-thalamic and fronto-parieto-cerebellar networks that mediate attention, inhibition, working memory, and timing (1). In addition to higher-order cognitive functions, abnormalities in sensorimotor processing and the default mode network have been identified in ADHD (1). ADHD affects approximately 5% of school-aged children and often persists into adolescence and adulthood (2). Consequently, patients with ADHD often experience impaired academic and social function, which increases the risk of other comorbidities including antisocial behavior, oppositional defiant disorder, and substance abuse (3). Hence, early diagnosis of ADHD and appropriate intervention are critical.

To date, administration of psychostimulant drugs, especially methylphenidate (MPH), has been a treatment of choice for ADHD children (4). However, in up to 30% of patients, MPH is ineffective or can be difficult to administer due to side effects such as insomnia, appetite loss, headaches, and irritability (5). Therefore, studies have been conducted to identify objective functional biomarkers by exploring the neural correlates of MPH effects (1). In these studies, various neuropsychological tests have been used to measure cognitive functions of interest in ADHD subjects. However, the utility of such computer-based tests for evaluating real-world performance is limited, because performance in a laboratory research setting may lack real-world translation. Indeed, traditional computerized neuropsychological tests are criticized for their lack of ecological validity. Similarly, there has been limited progress in research on how psychostimulants operate in real-life environments.

To overcome these limitations, the utility of virtual reality (VR) has been recognized in terms of diagnosis and treatment of ADHD. VR facilitates the creation of dynamic, immersive environments with three-dimensional stimuli in which attention can be tested in an environment comparable to that experienced in the real world, improving ecological validity (6). In the field of assessment, numerous studies have confirmed that continuous performance tests embedded in VR (VR-CPT) are as sensitive and accurate as traditional CPT (7–9). In addition, the efficacy of MPH has been verified through VR-CPT (10) and the effectiveness of VR rehabilitation programs, such as VR neurofeedback or cognitive training, have been demonstrated (6, 11). In a VR environment, training motivation is fostered by providing real-life contexts that connect cognitive training to goals of everyday life.

However, few studies have evaluated brain activity during VR tasks in ADHD patients. One of the reasons for the paucity of research on brain activity during VR tasks is due to difficulties in examination. It is physically challenging to perform functional magnetic resonance imaging (fMRI) or single-photon emission computed tomography (SPECT) scanning while using VR devices, especially for young ADHD patients with motor restlessness. Moreover, research on drug effects requires repeated pre- and post-administration imaging, which necessitates employment of a simple and easy tool to measure brain function. In this context, research using functional near-infrared spectroscopy (fNIRS) has been growing. fNIRS is a non-invasive imaging technique that uses near-infrared light to measure functional brain activity through changes in concentration of oxygenated hemoglobin (HbO) and deoxygenated hemoglobin (Hb). The main advantage of fNIRS over other functional neuroimaging modalities such as fMRI, SPECT, or magnetoencephalography (MEG) is its portability (12). The fMRI, MEG, and SPECT involve the use of large-sized equipment and the patient need to be in the supine position (13). In contrast, fNIRS facilitates the investigation of brain activation in ecologically valid settings as well as repetitive measurements with low-cost, safe, transportable instrumentation in the natural position (14). Furthermore, it is less sensitive to movement artifacts and shows greater spatial resolution than EEG. However, fNIRS can provide information on only cortical activity (13). Its inability to provide information on subcortical levels and cortical-subcortical connectivity can limit its use in studies on psychiatric disorders.

Nevertheless, over the last few years, fNIRS has been used extensively to investigate cortical alterations in patients with various psychiatric disorders, such as schizophrenia; mood, anxiety, and eating disorders; and substance use disorder (13). For example, using fNIRS, Kawakubo et al. (2009) found bilateral prefrontal cortex (PFC) hypoactivation during the verbal fluency task in patients on the autism spectrum disorder (15). In addition, it has been used to measure the therapeutic effect and efficacy of treatment and to identify life-time brain function development in patients with neurodevelopmental disorders such as ADHD to neurodegenerative disorders (13). Considering these previous studies and the advantages mentioned above, fNIRS is seen as a particularly suitable tool for evaluating participants who experience unavoidable movements, such as ADHD patients in clinical settings (16).

Various studies using fNIRS have been conducted in ADHD patients. In most studies using tasks for evaluating attention or inhibition, ADHD patients exhibited lower prefrontal activity than that of healthy control (HC) subjects during the task (17–20) although the results of studies using working memory tasks have been equivocal (21, 22). Furthermore, many studies have investigated the hemodynamic responses of MPH treatments using fNIRS, demonstrating that reduced right inferior frontal gyrus (IFG) and middle frontal gyrus (MFG) activation in ADHD patients compared to HCs was normalized after single-dose MPH administration (23, 24). These results indicate that the neural correlates of MPH effects can be verified with fNIRS.

However, there has been limited research investigating the effects of medication on brain activity using fNIRS in VR tasks which simulates real-life classroom settings. As cognitive skills are ultimately applied in the classroom setting, especially in school aged children, attention and executive function can be better encapsulated in the virtual classroom environment than using traditional computerized neuropsychological tests. Furthermore, previous studies used the Go/No-go and oddball tasks mainly to explore inhibitory function. However, inhibition alone is insufficient to explain the pathophysiological mechanisms of ADHD. Working memory (WM) impairment is considered a core deficit of ADHD associated with prefrontal dysfunction.

WM is defined as the ability to maintain information in an easily accessible state for a short period of time. According to the state-based model, the working memory contents are determined by perceptual & long-term memory representations being in a particular state of accessibility, maintained by neural activities controlled by the attentional processes (Supplementary Figure 1) (25). Information maintenance, an important element of working memory, is regulated by well-operated attentional processes, established by persistent neural activities in the relevant working areas (26). WM plays an important role in rapid processing and attention, and in turn, a large attention span and fast processing speed promote WM (27, 28). Rather than being determined by single brain region, WM appears to depend on good synchronization with the PFC and other brain areas, for example, the parietal cortex (27). In this process, the PFC is responsible for covering task-relevant information and for organizing fronto-parietal activity for sustained attention (29). For efficient activation of the WM network during task execution, the default mode network (DMN), which is activated in baseline cognitive state, performs deactivation coupling (30–32). The DMN is a large-scale brain network that consists of the core region of the medial prefrontal cortex (mPFC) and the postural cingulate/precuneus, along with inferior parietal lobule, lateral temporal cortex, and hippocampus (33). The DMN is largely related to mind wandering, which has known to affect performance of classroom of ADHD patients (34, 35). WM also plays an important role in maintaining focused behavior and improving classroom performance (36).

The N-back task is a well-known working memory paradigm which has been used extensively in functional neuroimaging studies on ADHD (37). A few studies investigated cortical brain activation using fNIRS during the n-back task. Herff et al. (2014) have found that different n-back conditions can be distinguished throughout fNIRS with high accuracy by the changes in hemodynamic response depending on the mental workload (38). Other studies using fNIRS also identified lower prefrontal complexity in patients with ADHD compared to in healthy control during the n-back task (39). However, few studies have explored the effects of MPH in relation to working memory using fNIRS in children with ADHD.

Based on these findings, we aimed to investigate the acute effects of MPH on behavioral performance and brain activity of children with ADHD during a VR n-back task in a virtual classroom setting. To the best of our knowledge, this is the first study using a VR-based working memory task and fNIRS to explore the effects of MPH in ADHD children.

## Methods

### Subjects

Altogether, 23 right-handed Korean children with ADHD (age range, 7–16 years; mean age, 9.96 ± 2.82) and 12 healthy control (HC) children (age range, 7–14 years; mean age, 11.33 ± 2.93) participated in this study. The number of participants required for adequate statistical power was based on previous studies that investigated drug effects in ADHD patients through fNIRS (23, 40–42) and a previous study that described the optimal design for functional brain imaging (43). The detailed demographic and clinical characteristics of subjects are listed in Table 1. ADHD patients were recruited by posting a notice on the outpatient clinic of Gangnam Severance Hospital, and the HC were recruited by posting announcements to the local internet community. All participants were interviewed by a psychiatrist to confirm the ADHD diagnosis according to the Diagnostic and Statistical Manual of Mental Disorders, 5th edition (DSM-5) (44). The Mini-International Neuropsychiatric Interview for Children and Adolescents (MINI-KID 6.0) was administered to all participants by certified psychologists (45). Exclusion criteria for participants with ADHD were clinically significant medical or neurological disorders, developmental disabilities including autism spectrum disorder, intellectual disabilities, speech impairments, severe learning disabilities, schizophrenia, bipolar disorder, substance and/or alcohol use disorders, IQ <70, or illiteracy to read consent. Participants in the HC group were excluded if they had current or past history of mental illness, clinically significant medical disease or neurological deficits, IQ <70, and/or illiteracy to read consent. IQ was measured with the Wechsler Intelligence Scale for children (WISC-IV).

**Table 1:** Demographic and clinical characteristics by group, comprising attention deficit hyperactivity disorder (ADHD) vs. healthy control (HC) subjects.

	**ADHD (*n* = 23)**	**HC (*n* = 12)**		
	**Mean (SD)**	**Mean (SD)**	**X2/t**	***p***
Age (years)	9.96 (2.82)	11.33 (2.93)	1.353	0.185
% Female	30.43	50	1.293	0.255
FSIQ	105.6 (12.81)	107.92 (10.6)	0.527	0.602
ADHD-RS-IV total	20.96 (14.51)	6.08 (3.68)	−4.64	<0.001^***^
ADHD-RS-inattention	12.3 (7.59)	4.25 (2.53)	−4.62	<0.001^***^
ADHD-RS-hyperactivity-impulsive	8.65 (7.55)	1.83 (1.7)	−4.137	<0.001^***^
CBCL total	62.41 (9.92)	49.08 (6.71)	−4.15	<0.001^***^
CBCL attention problem scores	64.41 (12.14)	52.58 (3.78)	−4.21	<0.001^***^
CDI	8.52 (6.1)	10.25 (7.85)	0.72	0.476
STAI-C				
STAI-C trait	29.09 (6.87)	30 (6.59)	0.378	0.708
STAI-C state	28.09 (8.96)	30.83 (7.04)	0.921	0.364
SSQ	18.14(22.32)	39.1(36.77)	1.983	0.057
MPH dose (mg)	29.35 (16.23)			
Comorbidity				
Tic disorder	*n* = 1			

*ADHD, attention deficit hyperactivity disorder; HC, Healthy Control; SD, standard deviation; FSIQ, full scale intelligence quotient; ADHD-RS, ADHD rating scale; CBCL, child behavior checklist; CDI, children's depression inventory; STAI-C, state-trait anxiety inventory for children; SSQ, simulator sickness questionnaire; MPH, methylphenidate*.

*** *p < 0.001*.

Several measurements were conducted to assess the psychological state of participants. The ADHD Rating Scales (ADHD-RS), an 18-question parent rating scale, was used to identify the presence of ADHD in children. Behavioral problems were assessed with the Korean version of the Child Behavior Checklist (CBCL), an 118-item parent-rated scale which queries behavioral problems in the past 6 months (46); Children's Depression Inventory (CDI) (47), a 27-item self-report scale to assess depressive symptoms (48); and State-Trait Anxiety Inventory for Children (STAI-C), a 20-item self-report scale were used to measure anxiety symptoms (49, 50). Simulator Sickness Questionnaire (SSQ), a 16-item self-report questionnaire was used to assess participants' subjective discomfort (disorientation, oculomotor symptoms, and nausea) after exposure to VR programs to measure simulator sickness due to discrepancies between vision and motion after VR use (51).

The protocol used for this study was approved by the Institutional Review Board of Yonsei University College of Medicine Gangnam Severance Hospital. Written informed consent and assent were obtained from all participants and one of their parents.

### Procedure

**Figure 1:**
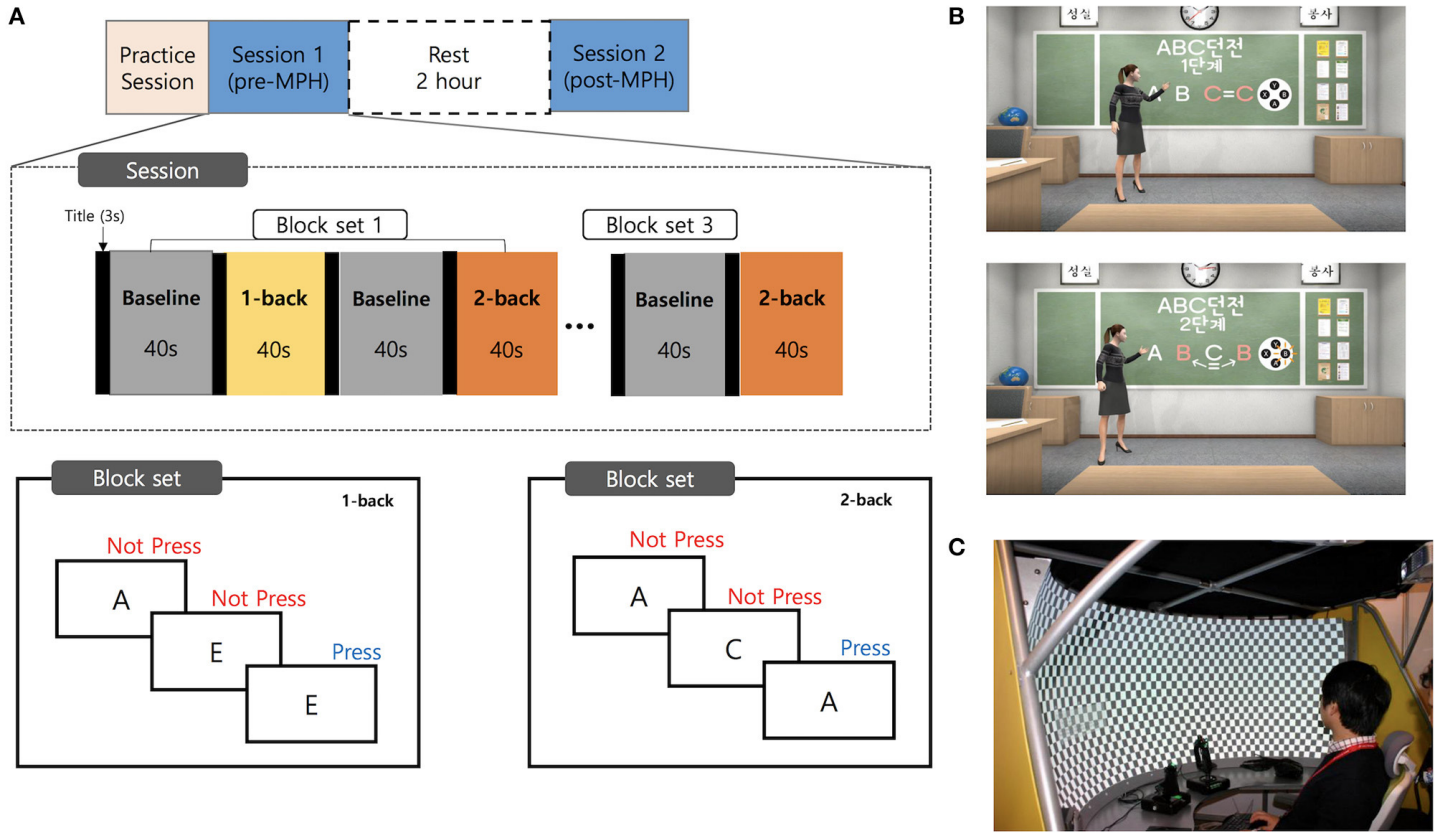
Experimental design and virtual reality environment. **(A)** A schematic representation of the flow of pre- and post-MPH n-back task sessions. **(B)** Screenshots of the virtual classroom during introduction of the n-back task. **(C)** The virtual reality theater with the curved screen. MPH, methylphenidate.

We investigated the effects of ADHD medication in a controlled pre- and post-MPH study design, whereby participants performed n-back tasks with the 1-back and 2-back conditions. The experimental protocol is summarized in Figure 1A. Participants underwent a medication washout period of 2 days before the examination. On the examination day, participants were first assessed for demographic, clinical characteristics, and IQ. Participants subsequently performed a practice session to familiarize themselves with the VR environment and tasks. Participants then underwent two test sessions, one before dosing and the other 2 h after dosing. After the first session, MPH (Concerta, Metadate or Medikinet) was administered orally. Experimental doses were the same as the participants' regular dose. Each test session was conducted in the order of the introduction session followed by an n-back task. The HC performed only one test session after the practice session.

### Virtual Reality Environment

The background of this VR task was a virtual classroom. The participants began by finding themselves in a typical classroom in a Korean school with desks facing ahead and blackboards in front of them. The participants' point of view was of the first person, facing the teacher, with other avatar classmates seated nearby (Figure 1B). The message “Please feel free to look around before class” offered the participants 15 s to adjust to the VR environment. A teacher avatar entered and informed participants of the rules of the N-back task, introduced as a game called “ABC Dungeon.” The participants first carried out a practice task, called “game tutorial” comprising 10 stimuli for each condition. The practice session was repeated if the participants made more errors than the set standard. Before the actual task, the teacher avatar briefly explained the fNIRS device, and participants were encouraged to remain as still as possible. After receiving instructions, participants were guided to wear fNIRS gear, followed by the main task.

The n-back task consisted of three block sets. Each block set contained alternating 1-back (low WM load) and 2-back (high WM), and in-between 0-back (control) conditions (block length, 40 s; 20 trials with random display of capitalized letters from “A” to “G”). Each stimulus was presented for 500 ms with an interstimulus interval of 1.5 s. Overall block-set time was 160 s, and total session time was 8 min. The total number of trials in experimental conditions was 120. Subjects were instructed to press the button with their right index finger as quickly as possible, when the current stimulus was the same as the previously shown letter (1-back), or with the letter shown two screens back (2-back). Since it is extremely difficult for young ADHD patients to remain still when not performing a task, our baseline (control) task required participants to respond to each stimulus with a button press to rule out motion artifacts and equalize the motion load with the experimental task. To diminish habituation or practice effects in the post-MPH session, two task versions with different stimuli were employed. These two versions were randomly assigned to each subject.

The software was written in Visual Studio 2017(C#) and designed on Unity 2018.2. The avatars and structures comprising the virtual environment were built using a 3Ds Max 2014. We used the 3D development platform (Vizard 5.1; WorldViz, Santa Barbara, CA, USA) to develop the virtual classroom environment. Our VR classroom was implemented in the VR theater which provides a semi-immersive environment with a 2.18-m radius curved screen, providing 150° field-of-view (PACOM Display System Inc, Suwon, Kyungki-do, Republic of Korea; Figure 1C). Two projectors with HDTV resolution (1,960 × 1,080 pixels) were used to project the programs onto the screen. The system was driven by a desktop computer with Microsoft Windows 10 operating system, including a high-end graphics card (NVIDIA GeForce GTX 970) and 16 GB RAM of graphics memory.

### fNIRS Measurements

**Figure 2:**
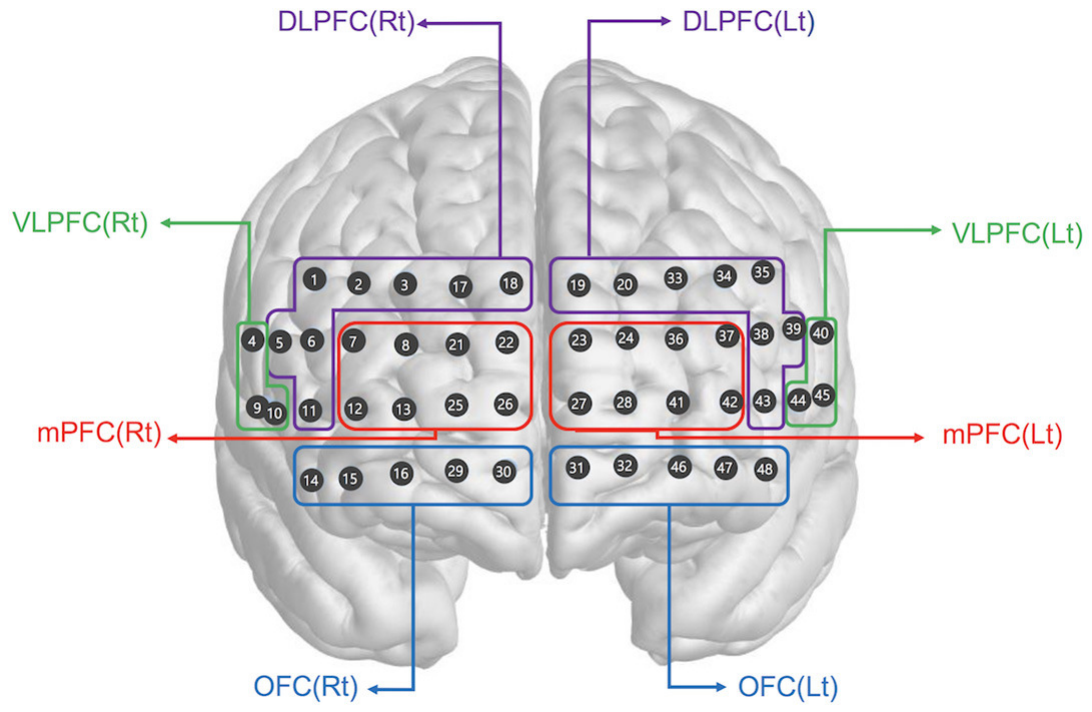
Spatial profiles of fNIRS channels. The fNIRS probes were attached to the prefrontal area. The included channels are represented according to brain region location. DLPFC, dorsolateral prefrontal cortex; VLPFC, ventrolateral prefrontal cortex; mPFC, medial prefrontal cortex; OFC, orbitofrontal cortex; Rt, right; Lt, left.

**Figure 3:**
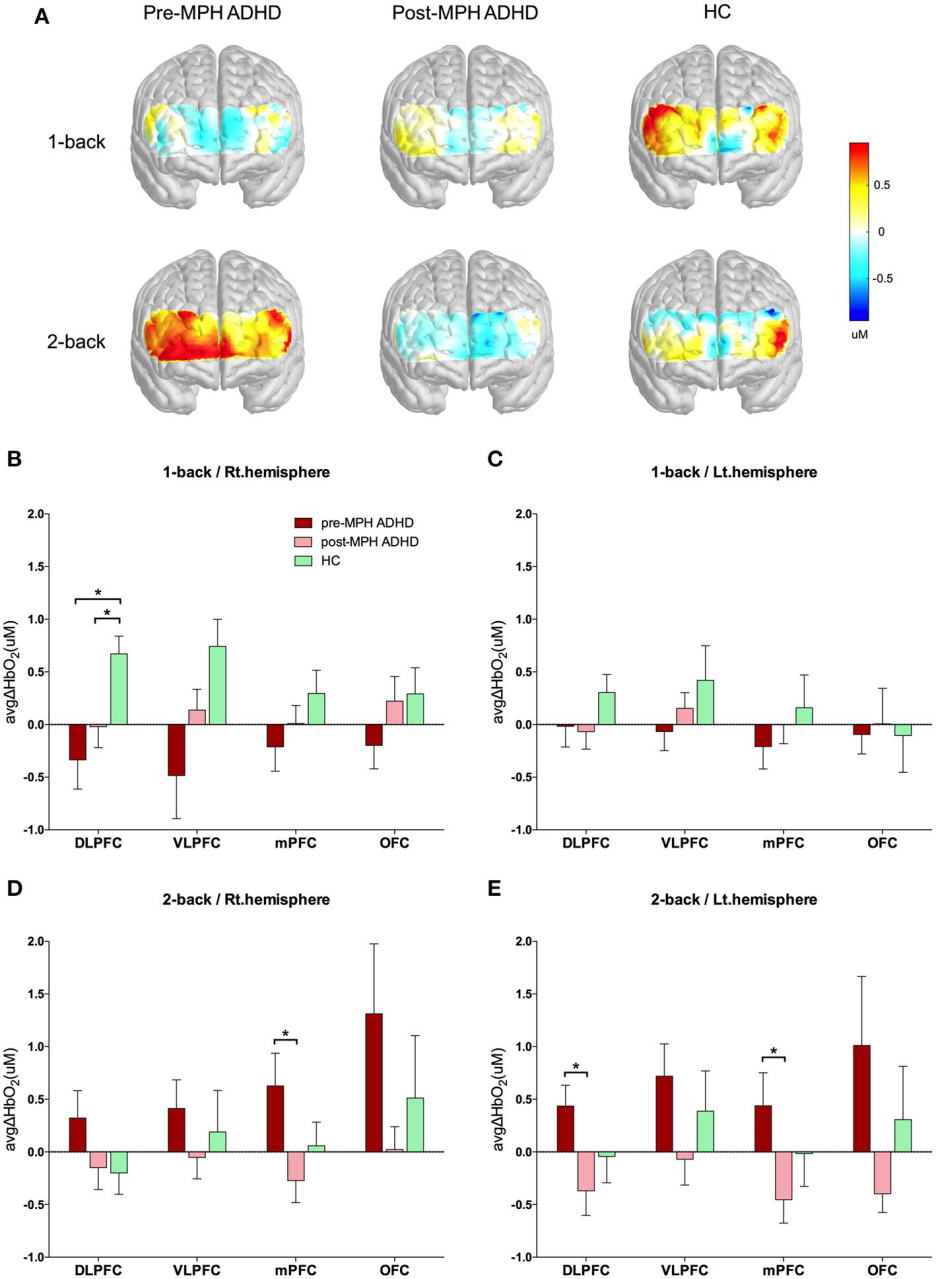
Hemodynamic changes during performance of the n-back task. **(A)** The overall brain activation patterns are shown as signal maps with avgΔHbO_2_ values presented in accordance with the color bar. The upper and lower lines show results for 1-back and 2-back condition, respectively, of each group. The mean avgΔHbO_2_ values for 1-back condition in **(B)** right and **(C)** left hemispheres and 2-back condition in **(D)** right and **(E)** left hemispheres are represented as bar graphs. ADHD, attention deficit hyperactivity disorder; HC, healthy control; MPH, methylphenidate; Hb, hemoglobin; DLPFC, dorsolateral prefrontal cortex; VLPFC, ventrolateral prefrontal cortex; mPFC, medial prefrontal cortex; OFC, orbitofrontal cortex; Rt, right; Lt, left. *p < 0.05.

We used a multichannel high density fNIRS device (NIRSIT; OBELAB, Seoul, Korea), which consisted of 24 laser diodes emitting two wavelengths (780/850 nm) and 32 photodetectors separated by a 1.5 cm unit distance. The laser and detector pairs were separated at a 3 cm distance. Sampling rate was 8.138 Hz. The alignment of 48 channels is shown in Figure 2. The fNIRS device was placed on the head according to the relevant standard positions of the International 10–20 system for EEG electrode locations. The center of the bottom line of the measuring channel was located on the FPZ. The threshold of signal-to-noise ratio was 30 dB such that slow drift of physiological noise and environmental noise was removed after filtering through a band-pass filter (0.005–0.1 Hz) of detected light signals. The modified Beer Lambert Law (MBLL) was used to convert raw light intensities into concentration changes in oxygenated hemoglobin (ΔHbO_2_). The averaged oxy-Hb concentration changes (avgΔHbO_2_) during the task period baselined from 5 s before task initiation was calculated in each channel after block averaging of multiple trials. Finally, the regional representative value of avgΔHbO_2_ was extracted by averaging categorized channels based on the specified region of interest (ROI). The selection of the brain ROI was completed before data analysis. The 48 channels were categorized as right and left dorsolateral prefrontal cortex (DLPFC), ventrolateral prefrontal cortex (VLPFC), medial prefrontal cortex (mPFC), and orbitofrontal cortex (OFC), which constituted eight ROIs. The channels corresponding to each region are shown in Figure 2. The MNI coordinates for each channel were defined based on the equipment coordinates. Using this information, the ROIs were designated in accordance with the Brodmann area template for each channel. Brain activation maps (Figure 3A) were visualized using the avgΔHbO_2_ per channel of each group according to 1-back and 2-back tasks.

### Behavioral Data Analysis

For n-back task results, the total reaction time (RT), RT variability, and accuracy were used as dependent variables for analysis. RT variability was calculated by dividing the standard deviation of the individual RT by the mean value, as reported previously (52). We computed accuracy in each condition by dividing the correct answer (correct response and appropriate rejection) by total number of stimuli.

### Statistical Analysis

Group differences in clinical characteristics between ADHD and HC groups were compared using an independent sample *t*-test for numerical variables or Chi square (χ^2^) test for categorical variables. The means and standard deviations of avgΔHbO_2_ were calculated for each ROI and in each group to compare group differences and verify the effects of pre- and post-MPH effects on fNIRS results. For comparison of behavioral performance and fNIRS data between pre- and post-treatment conditions in ADHD participants, we used a two-tailed paired *t*-test. An independent two-sample two-tailed *t*-test was used for comparing variables between ADHD and HC groups. The normality of the data was evaluated by visual inspection of quantile-quantile plots and the Shapiro-Wilk test. To examine the association between behavioral performance and fNIRS data, we conducted correlation analysis using the Pearson's method. The statistical threshold was set at *p* < 0.05. All statistical analyses were completed with IBM SPSS version 25 (IBM Corporation, Armonk, NY, USA).

## Results

### Demographic and Clinical Characteristics

Baseline characteristics of the study participants are presented in Table 1. ADHD and HC groups did not differ significantly in mean age, sex ratio, full scale IQ(FSIQ), CDI, STAI-C, or SSQ scores (Table 1). With regard to comorbidities, only one patient with ADHD had a tic disorder. Both ADHD patients and HC showed lower CDI and STAI-C scores than the cut-off (ADHD, CDI = 8.52, STAI-C trait = 29.09, STAI-C state = 28.07; HC, CDI = 10.25, STAI-C trait = 30, STAI-C state = 30.83; cut-off, CDI = 13, STAI-C trait = 36, STAI-C state = 36), indicating the absence of clinical depression and anxiety. As expected, significant differences were observed in the ADHD-RS (*t* = −4.64, *p* < 0.001), ADHD-RS-inattention and hyperactive-impulsive subscales scores (inattention, *t* = −4.62, *p* < 0.001; hyperactive-impulsive, *t* = −4.137, *p* < 0.001), CBCL total and attention problem subscale scores (total, *t* = −4.15, *p* < 0.001; attention problem, *t* = −4.21, *p* < 0.001). The mean ADHD_RS score of the ADHD patients was 20.96, which indicates relatively mild level of ADHD severity. The SSQ score was low in both groups (ADHD, 18.14; HC, 39.31) with few symptoms. The typical mean MPH dose of ADHD subjects was 29.35 mg (SD, 16.23 mg; range, 10–64 mg).

### Behavioral Performance

The average accuracy rates and RTs in each n-back task for HC and ADHD participants are summarized in Table 2. Within-ADHD-subject analysis revealed shorter RTs in the 1-back condition and shorter RT, lower RT variability, and higher accuracy in the 2-back condition after MPH administration compared to that of pre-MPH. No significant differences in behavioral performance between conditions were observed for ADHD and HC groups.

**Table 2:** N-back task performance for ADHD and control subjects.

	**ADHD**										**HC**	
	**Pre-MPH vs. post-MPH**		**Pre-MPH**		**Pre-MPH vs. HC**		**Post-MPH**		**Post-MPH vs. HC**			
	***t***	***p***	**Mean**	**SD**	***t***	***p***	**Mean**	**SD**	***t***	***p***	**Mean**	**SD**
**1-back**												
RT (ms)	3.15	0.005^**^	677.63	146.68	−1.159	0.255	602.83	119.84	0.118	0.907	609.18	189.61
RT variability (SD/mean)	1.26	0.221	0.31	0.14	−0.525	0.603	0.26	0.13	0.419	0.597	0.28	0.11
Accuracy (correct %)	−1.691	0.106	92.35	13.22	−0.05	0.961	95.07	9.32	−0.827	0.415	92.13	10.68
**2-back**												
RT (ms)	2.58	0.018^*^	792.14	160.09	−1.512	0.141	695.66	149.26	0.281	0.78	710.16	128.98
RT variability (SD/mean)	3.005	0.007^**^	0.46	0.16	−1.271	0.213	0.34	0.11	1.375	0.179	0.39	0.1
Accuracy (correct %)	−3.158	0.005^**^	82.33	12.2	0.869	0.391	87.59	12.18	−0.362	0.72	86.05	11.09

*RT and accuracy are presented for each condition. For ADHD subjects, data for pre-, post-MPH, and pre-MPH minus post-MPH are presented. T-values, p-values, and statistical significance in pre- and post-MPH columns are the results of Student's t-test between HC and each ADHD condition. Those in the pre- vs. post-MPH column are the results of a paired t-test. ADHD, attention deficit hyperactivity disorder; HC, healthy control; MPH, methylphenidate; SD, standard deviation; RT, reaction time*.

* *p < 0.05*,

** *p < 0.01*.

### fNIRS Results

Changes in avgΔHbO_2_ measured using fNIRS are presented in Figure 3 according to brain area and group. For the (low WM load) 1-back condition, no significant avgΔHbO_2_ changes were observed in any area when comparing pre- and post-MPH conditions (Figures 3B,C; Supplementary Table 1). Compared to the HC group, both pre-MPH and post-MPH ADHD subjects showed significantly fewer avgΔHbO_2_ signals in the right DLPFC (pre-MPH vs HC, *t* = −2.443, *p* < 0.05; post-MPH vs. HC, *t* = −2.233, *p* < 0.05; Figure 3B; Supplementary Table 1). For the (high WM load) 2-back condition, avgΔHbO_2_ in pre-MPH ADHD subjects was significantly higher than that in post-MPH ADHD subjects in the left DLPFC and bilateral mPFC (left DLPFC, *t* = 2.838, *p* < 0.05, uncorrected; left mPFC, *t* = 2.334, *p* < 0.05, uncorrected; right mPFC, *t* = 2.496, *p* < 0.05, uncorrected; Figures 3D,E; Supplementary Table 1). No significant differences were observed when comparing the HC group with both pre- and post-MPH ADHD subjects. In the 2-back task, decreased activation was noted in several brain areas after MPH administration in ADHD subjects.

### Association Between Behavioral Performance and fNIRS Results

**Figure 4:**
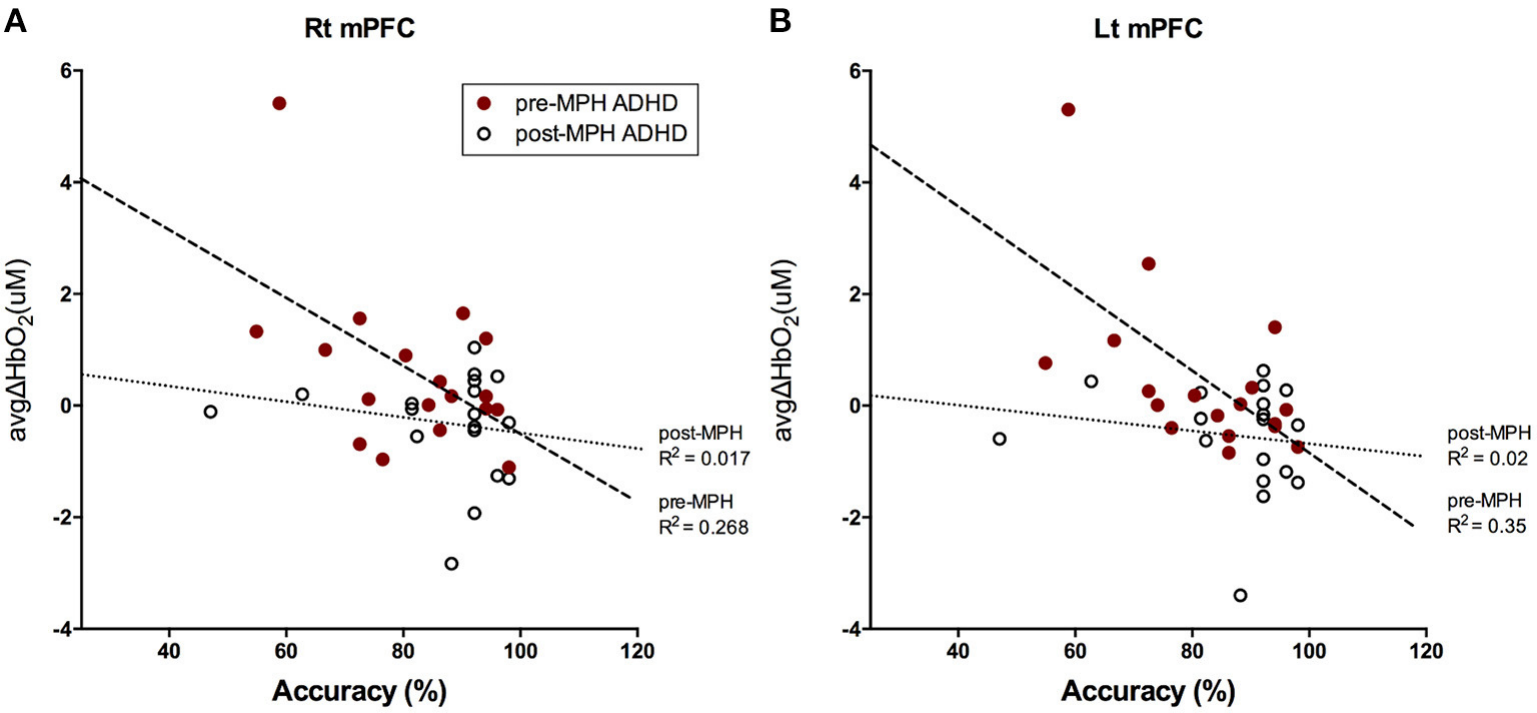
Correlation between 2-back task accuracy and avgΔHbO_2_ signals in bilateral mPFC. Correlations between accuracy (%) for 2-back task and avgΔHbO_2_ values in **(A)** right mPFC and **(B)** left mPFC in pre-(red dot) and post-MPH (white dot) ADHD subjects were analyzed with Pearson's correlation test. Line and R2 value indicate the regression of the dataset (dashed, pre-MPH ADHD; dotted, post-MPH ADHD).

We investigated the relationship between 2-back task accuracy (% correct) and mPFC activation. There was a negative correlation between accuracy and avgΔHbO_2_ in the right and left mPFC in pre-MPH ADHD subjects (right mPFC, *r* = −0.518, *p* < 0.05; left mPFC, *r* = −0.591, *p* < 0.05; Figures 4A,B). After MPH administration, this correlation was not observed.

## Discussion

The present study aimed to investigate the feasibility of using fNIRS to investigate the neural substrates of single-dose MPH effects in children with ADHD during a VR working memory task. The combination of VR and fNIRS in this study revealed that this model was able to easily measure the effects of various drugs and interventions on children with ADHD in an environment simulating a real-world setting. Furthermore, it enables a setting that explores changes in brain activity during a neuro-rehabilitation program using VR environments. To our knowledge, this is the first study using a VR environment to measure the impact of MPH on working memory with fNIRS in ADHD subjects.

In the current study, task performance improved after MPH treatment; this was verified by shorter RT in the 1-back condition and shorter RT, lower RT variability, and higher accuracy in the 2-back condition compared to that in pre-medicated ADHD subjects. This result is generally consistent with previous studies reporting performance enhancing effects of MPH in ADHD patients (53–55). Conversely, there was no performance differences between pre- and post-medicated ADHD subjects and the HC group in the VR n-back task. This result was also reported in our previous study that examined VR-CPT performance in ADHD and HC subjects. In this study, scores were lower in the ADHD group than in the HC group in traditional CPT, but VR-CPT performance was not significantly different. Similarly, it is possible that VR increased the motivation of ADHD patients by presenting them with a more immersive environment in the current study, thereby improving their task performance, as reported in our previous VR study (56). Although the enjoyment or motivation of patients was not measured in this study, previous studies have shown that VR neuropsychological tests were perceived as more enjoyable to patients and increased motivation (9, 57). For precise interpretation, further research is needed on motivation and performance differences in ADHD patients when using VR.

We observed increased activation in the left DLPFC and bilateral mPFC in pre-medicated ADHD subjects during the VR 2-back task, which decreased after MPH administration. However, there were no significant differences between HC and ADHD children. In most NIRS studies comparing children with ADHD to HCs in different executive functioning tasks, altered prefrontal activity was reported: some studies reported reduced activity in ADHD (17–19), whereas others reported increased activity in ADHD (58, 59). The cause of these conflicting results is not clear, but a possible reason is the use of different cognitive tasks with different stimulus characteristics. Therefore, the brain areas associated with task performance in each study were not concordant, rendering the neural correlates of WM in ADHD subjects unclear. Previous meta-analyses revealed significant hypoactivation in the left inferior frontal gyrus/anterior insula and right middle frontal gyrus (60), bilateral superior frontal gyrus, and left medial frontal gyrus (61) in adult ADHD patients during the N-back task compared to that in HCs. Nevertheless, meta-analyses on the neural substrates related to WM in children with ADHD are limited. One meta-analysis on healthy children found no concordance in prefrontal regions related to the n-back task (62). In addition, the only study exploring the effects of MPH on brain function during a WM task reported that no specific brain area was activated by stimulants (54). These discrepancies may be due to varying brain maturity and subjective n-back difficulty according to age (62). Further, medication status may differentially affect brain activation. A previous study reported that medication-naïve ADHD patients showed a tendency toward lower activation than that of HCs, whereas non-naïve and HCs did not differ significantly but showed similar activation (42). Given these conflicting results, it is difficult to conclude whether our findings agree with the literature.

Increased activation in bilateral mPFC was negatively correlated with accuracy of the 2-back task in pre-medicated ADHD subjects, whereas this association disappeared after MPH treatment. This result implies that mPFC was abnormally hyper-activated in ADHD subjects during the 2-back task, which was accompanied by low performance. The mPFC is a brain region that constitutes the default mode network (DMN) along with the medial and lateral parietal and temporal cortices (63). The DMN is associated with intrinsic brain activity and commonly deactivates during attention-demanding or goal-directed activity. Abnormal hyperactivity in DMN areas, indicating unsuccessful task-induced deactivation, is characteristic of ADHD patients (55, 64, 65). Impairment of sustained attention in ADHD may result from abnormal persistence or intrusion of DMN activity (64). In addition, several studies reported that psychostimulants normalized abnormal hyperactivity in the DMN, including the mPFC, in ADHD subjects, and even in children (55, 65, 66). Our study also demonstrated that MPH administration reduced mPFC activation while improving task performance, which indicated normalization of attenuated DMN deactivation in ADHD children. However, a double-blind placebo-controlled study involving other areas comprising the DMN should be performed to understand the relevance of these findings.

By contrast, in the 1-back condition, we found no difference in brain activation between pre- and post-MPH conditions. Given that only RT was shorter after MPH treatment for behavioral performance, and there was no significant difference in accuracy or RT variability, it can be assumed that the difference before and after drug was minimal due to the low level of 1-back difficulty, reflected by a ceiling effect from the pre-test. The only study that explored the effect of MPH during the n-back task in ADHD children reported no differences in brain function in the 1-back condition between pre- and post-drug administration, which is concordant with our study (67). In addition to exploring MPH effects in a VR environment, brain activity was also measured with fNIRS in this study in a VR-based WM task. Compared to the HC group, ADHD children showed lower activation of the right DLPFC, consistent with previous studies on ADHD and HC participants in which the right DLPFC was a ROI identified via fNIRS (37).

In the present study, the VR classroom environment provided a more ecologically valid and motivating task than that of traditional computerized cognitive tasks, suggested by the improved task performance of ADHD children. We propose several advantages of using a VR task over traditional computerized WM tasks. Previous studies have indicated that ADHD children find digital technology environments more enjoyable and are more immersed in the task (9, 68). None of the participants complained of side effects in SSQ, suggesting that the virtual classroom is a user-friendly tool. This may be regarded as an alternative for estimating how brain activity changes with WM in ADHD children after drug administration in a setting that simulates real-world classrooms.

### Limitations

Our study has several limitations which we hope to address in future studies. First, the study design was not optimized for neuropharmacological analysis. A double-blind placebo-controlled or a cross-over design with drug-naive ADHD children should be conducted. Since we did not include a placebo group, the superior task performance in post-MPH ADHD subjects could be due to a repeat effect. In addition, unlike the ADHD patients, the HC did not repeat the tests, making it difficult to compare the HC group with the post-MPH group or to eliminate the time effect. Therefore, it is not entirely clear whether the changes in brain activation in the ADHD group were truly induced by the treatment or were simply a function of time. However, considering the NIRS imaging results, the improved behavioral performance was accompanied by changes in brain activity such as reduced hyperactivity of bilateral mPFC in post-MPH ADHD subjects, making it difficult to fully explain solely based on repeat effects. Further, WM tasks are generally considered to have strong test-retest reliability and to be relatively unaffected by practice effects (69). In addition, previous studies suggested that the effects of MPH on brain activity differ between drug-naive and non-naive ADHD subjects (42, 70, 71). Although our subjects may have exhibited weakened pure drug effects because they were a drug-non-naive group, it is relevant for feasibility because it is necessary to measure drug effects in non-naive subjects in real clinical practice. Further studies including both drug-naïve and non-naïve children could lead to accurate interpretations. Second, due to the limitations in our experimental design, such as the relatively small sample size and lack of a cross-over design, a liberal statistical threshold was adopted for this study. Third, the relatively wide age range of the subjects (7–16 years) and the lack of sex stratification could be problematic. A meta-analysis of fMRI studies related to the n-back task revealed widespread variability of prefrontal activation patterns across ages due to a protracted, step-wise maturation pattern of the prefrontal cortex (62). Although most ADHD research is conducted with similar age ranges to ours, it will help to narrow down the age range to elicit more accurate results on prefrontal function in child participants. In addition, although previous studies have shown sex differences, such as hypofrontality only in males, we did not conduct a subgroup analysis by sex due to the small sample size of this study (72, 73). Future studies with large sample sizes to analyze group effects by age and sex are needed. Fourth, the brain regions evaluated in this study were not wide enough to cover WM-related areas or DMN. The fNIRS probe covered only the prefrontal cortex and was unable to detect activity in deeper cortical structures unreachable by near-infrared light. Further studies covering wider areas or exploring connectivity between brain areas are needed to clearly interpret the results of this study. Fifth, we cannot determine if the VR n-back task is actually more effective or motivation-enhancing because we lack a comparison of a VR paradigm to a 2D version of the n-back task. Finally, we used a semi-immersive VR display, instead of a more immersive HMD version of virtual reality, since simultaneously wearing HMD and fNIRS devices is difficult due to space overlap.

## Conclusions

To our knowledge, this is the first study to explore the effects of MPH on brain activity during a VR-based WM task reproducing real-classroom settings in ADHD children. We observed that activation of bilateral mPFC decreased after MPH treatment in a high-load WM task. Further, bilateral mPFC activation was negatively correlated with task accuracy in pre-MPH condition; this correlation disappeared after MPH administration. These findings suggest that mPFC-mediated inappropriately excessive mind-wandering during a high-load WM task in ADHD children may have disappeared after MPH administration. Taken together, these results suggest that the combination of VR tasks and fNIRS examination is a technique that enables examination of the effects of interventions within a real-life setting in ADHD children and adolescents.

## Data Availability Statement

The raw data supporting the conclusions of this article will be made available by the authors, without undue reservation.

## Ethics Statement

The studies involving human participants were reviewed and approved by Institutional Review Board of Yonsei University College of Medicine Gangnam Severance Hospital. Written informed consent to participate in this study was provided by the participants' legal guardian/next of kin. Written informed consent was obtained from the minor(s)' legal guardian/next of kin for the publication of any potentially identifiable images or data included in this article.

## Author Contributions

EK and NL devised the project, the main conceptual ideas, and proof of the outline. EK, NL, and JY designed the detailed study design. JY, NH, JK, and JH examined participants, performed the experiments, and acquired and organized the data. SJ and JC conducted analyses of the experimental data, drafted the manuscript, and designed the figures with supervision from EK. JO and J-jK contributed to interpreting the results and worked on the manuscript. All authors discussed the results and commented on the manuscript. EK supervised the project. All authors contributed to the article and approved the submitted version.

## Conflict of Interest

JC was employed by OBELAB Inc. The remaining authors declare that the research was conducted in the absence of any commercial or financial relationships that could be construed as a potential conflict of interest.
