# Supplementary material for: Use of Virtual Reality Working Memory Task and Functional Near-Infrared Spectroscopy to Assess Brain Hemodynamic Responses to Methylphenidate in ADHD Children
Source: Front Psychiatry. 2021 Jan 21;11:564618. doi: 10.3389/fpsyt.2020.564618 (PMC7859615; doi:10.3389/fpsyt.2020.564618)

Supplementary Material

**Supplementary Table 1**. Hemodynamic changes for ADHD and control subjects in n-back task.

|  | ADHD | | | | | | | | | | | | | |  | HC | |
| --- | --- | --- | --- | --- | --- | --- | --- | --- | --- | --- | --- | --- | --- | --- | --- | --- | --- |
|  | Pre-MPH vs post-MPH | |  | Pre-MPH | | | Pre-MPH vs HC | |  | Post-MPH | | Post-MPH vs HC | | |  |  | |
|  | t | p |  | Mean  (uM) | SD | t | | p |  | Mean  (uM) | SD | | t | p |  | Mean  (uM) | SD |
| *1-back* |  |  |  |  |  |  | |  |  |  |  | |  |  |  |  |  |
| Rt |  |  |  |  |  |  | |  |  |  |  | |  |  |  |  |  |
| DLPFC | -0.882 | .389 |  | -0.34 | 1.24 | -2.443 | | .021^*^ |  | -0.02 | 0.9 | | -2.233 | .034^*^ |  | 0.67 | 0.53 |
| VLPFC | -1.244 | .229 |  | -0.48 | 1.84 | -2.001 | | .055 |  | 0.14 | 0.88 | | -1.811 | .081 |  | 0.74 | 0.82 |
| mPFC | -0.906 | .376 |  | -0.21 | 1.03 | -1.394 | | .174 |  | 0.01 | 0.76 | | -0.993 | .329 |  | 0.3 | 0.7 |
| OFC | -1.613 | .123 |  | -0.2 | 0.99 | -1.357 | | .186 |  | 0.22 | 1.05 | | -0.182 | .857 |  | 0.3 | 0.79 |
| Lt |  |  |  |  |  |  | |  |  |  |  | |  |  |  |  |  |
| DLPFC | 0.186 | .855 |  | -0.02 | 0.88 | -1.053 | | .301 |  | -0.07 | 0.74 | | -1.398 | .173 |  | 0.3 | 0.55 |
| VLPFC | -0.893 | .383 |  | -0.07 | 0.81 | -1.398 | | .173 |  | 0.15 | 0.66 | | -0.854 | .4 |  | 0.42 | 1.04 |
| mPFC | -1.052 | .386 |  | -0.21 | 0.95 | -0.992 | | .33 |  | <-0.01 | 0.8 | | -0.483 | .633 |  | 0.16 | 0.98 |
| OFC | -0.314 | .757 |  | -0.09 | 0.83 | 0.028 | | .978 |  | -0.01 | 1.51 | | 0.182 | .857 |  | -0.1 | 1.11 |
| *2-back* |  |  |  |  |  |  | |  |  |  |  | |  |  |  |  |  |
| Rt |  |  |  |  |  |  | |  |  |  |  | |  |  |  |  |  |
| DLPFC | 1.503 | .149 |  | 0.32 | 1.17 | 1.314 | | .2 |  | -0.15 | 0.93 | | 0.154 | .878 |  | -0.2 | 0.64 |
| VLPFC | 1.349 | .193 |  | 0.41 | 1.21 | 0.47 | | .642 |  | -0.05 | 0.91 | | -0.603 | .552 |  | 0.19 | 1.25 |
| mPFC | 2.496 | .022^*^ |  | 0.63 | 1.39 | 1.212 | | .236 |  | -0.27 | 0.94 | | -0.98 | .335 |  | 0.06 | 0.71 |
| OFC | 1.81 | .086 |  | 1.31 | 2.97 | 0.775 | | .445 |  | 0.02 | 0.97 | | -0.949 | .351 |  | 0.51 | 1.87 |
| Lt |  |  |  |  |  |  | |  |  |  |  | |  |  |  |  |  |
| DLPFC | 2.838 | .011^*^ |  | 0.44 | 0.88 | 1.441 | | .161 |  | -0.37 | 1.04 | | -0.872 | .391 |  | -0.04 | 0.79 |
| VLPFC | 1.97 | .064 |  | 0.72 | 1.37 | 0.651 | | .52 |  | -0.07 | 1.09 | | -1.0 | .307 |  | 0.39 | 1.21 |
| mPFC | 2.334 | .031^*^ |  | 0.44 | 1.41 | 0.912 | | .37 |  | -0.45 | 0.99 | | -1.142 | .263 |  | 0.02 | 0.98 |
| OFC | 1.95 | .066 |  | 1.01 | 2.93 | 0.705 | | .487 |  | -0.04 | 0.8 | | -1.627 | .115 |  | 0.31 | 1.6 |

The avgΔHbO_2_ signals for each region and group are presented. For ADHD subjects, values for pre-, post-MPH, and pre-MPH minus post-MPH are shown. T-values, p-values, and statistical significance in pre- and post-MPH columns are the results of Student’s t-test between CTL and each ADHD condition. Those in the pre- versus post-MPH column are the results of a paired t-test. ADHD, attention deficit hyperactivity disorder; HC, healthy control; MPH, methylphenidate; SD, standard deviation; DLPFC, dorsolateral prefrontal cortex; VLPFC, ventrolateral prefrontal cortex; OFC, orbitofrontal cortex; mPFC, medial prefrontal cortex; Rt, right; Lt, left

**Supplementary Figure 1.** Simplified diagram of Cowan’s (1988) embedded-processes model, the most prominent representative of state-based models.


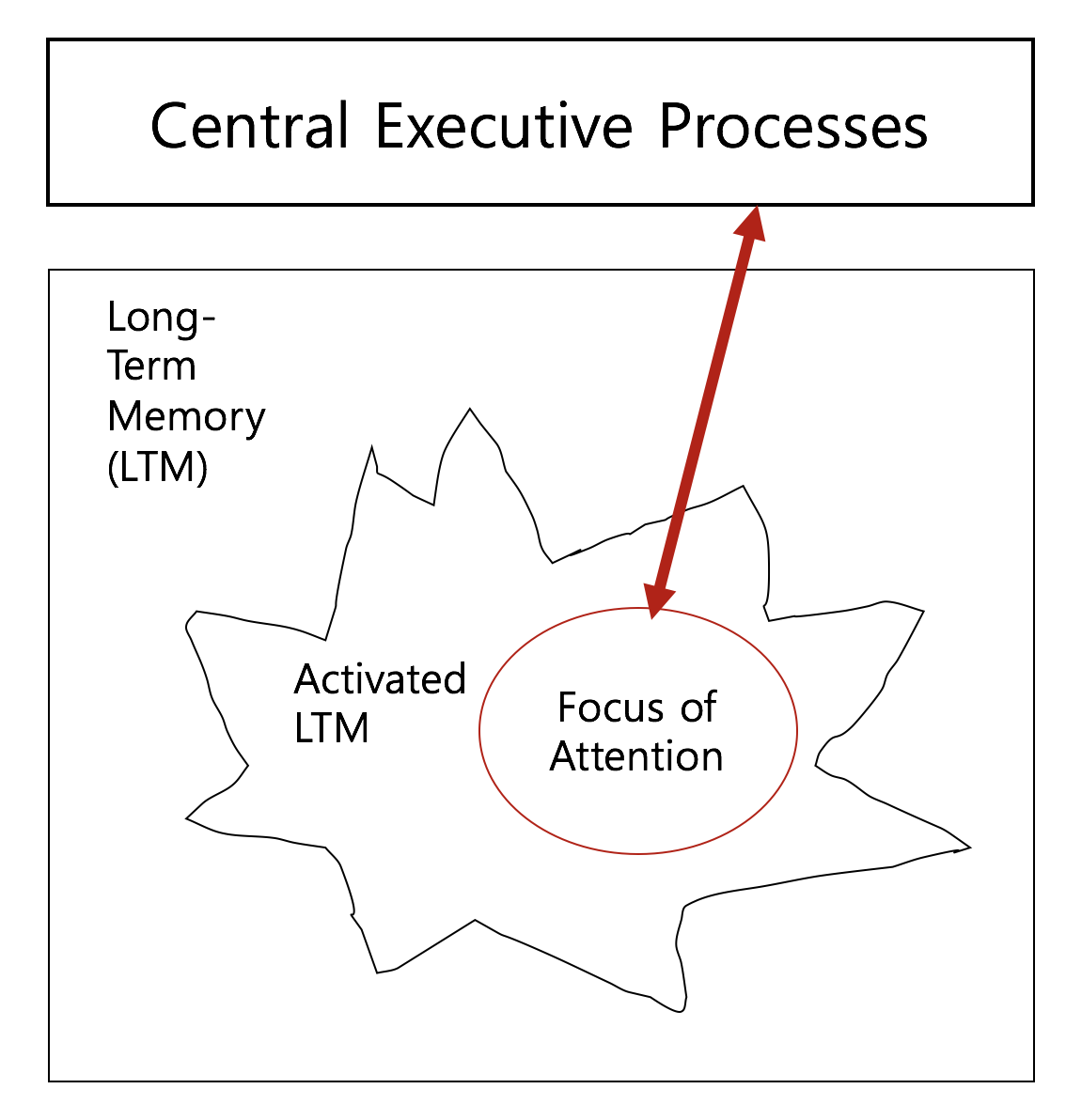

Supplement: Supplementary file 1 [file Data_Sheet_1.docx]
